# Health care personnel’s perspectives on human papillomavirus (HPV) self-sampling for cervical cancer screening: a pre-implementation, qualitative study

**DOI:** 10.1186/s43058-022-00382-3

**Published:** 2022-12-13

**Authors:** Serena Xiong, De Ann Lazovich, Faiza Hassan, Nafisa Ambo, Rahel Ghebre, Shalini Kulasingam, Susan M. Mason, Rebekah J. Pratt

**Affiliations:** 1 Department of Surgery, Washington University School of Medicine, 600 S Taylor Avenue, St. Louis, MO 63110 USA; 2 Department of Epidemiology and Community Health, University of Minnesota School of Public Health, 1300 S 2nd Suite 300, Minneapolis, MN 55454 USA; 3 Masonic Cancer Center, University of Minnesota, 425 East River Parkway, Minneapolis, MN 55455 USA; 4 Program in Health Disparities Research, University of Minnesota Medical School, 717 Delaware Street SE, Suite 166, Minneapolis, MN 55414 USA; 5 Department of Obstetrics, Gynecology and Women’s Health, University of Minnesota Medical School, 420 Delaware St SE, Minneapolis, MN 55455 USA

**Keywords:** Human papillomavirus self-sampling, Implementation, Qualitative research, Consolidated Framework for Implementation Research, Cervical cancer screening

## Abstract

**Background:**

Persistent infection with high-risk human papillomavirus (hrHPV) types is a well-documented cause of cervical cancer. Since the implementation of cervical cancer screening methods (e.g., Pap tests), cervical cancer rates have declined. However, Pap tests are still unacceptable to many women and require complex infrastructure and training. Self-sampling techniques for collecting HPV specimens (or “HPV self-sampling”) have been proposed as a possible alternative to overcome these barriers. The objective of this study was to capture perspectives from health care personnel (providers, leaders, and clinic staff) across primary care systems on the potential implementation of an HPV self-sampling practice.

**Methods:**

Between May and July 2021, a study invitation was emailed to various health care professional networks across the Midwest, including a snowball sampling of these networks. Eligible participants were invited to a 45–60-min Zoom-recorded interview session and asked to complete a pre-interview survey. The survey collected sociodemographics on age, occupation, level of educational attainment, race/ethnicity, gender, and awareness of HPV self-sampling. The semi-structured interview was guided by the Consolidated Framework for Implementation Research and asked participants about their views on HPV self-sampling and its potential implementation. All interviews were audio-recorded, transcribed, and analyzed using NVivo 12.

**Results:**

Key informant interviews were conducted with thirty health care personnel—13 health care providers, 6 clinic staff, and 11 health care leaders—from various health care systems. Most participants had not heard of HPV self-sampling but reported a general enthusiasm for wanting to implement it as an alternative cervical cancer screening tool. Possible barriers to implementation were knowledge of clinical evidence and ease of integration into existing clinic workflows. Potential facilitators included the previous adoption of similar self-sampling tools (e.g., stool-based testing kits) and key decision-makers.

**Conclusion:**

Although support for HPV self-sampling is growing, its intervention’s characteristics (e.g., advantages, adaptability) and the evidence of its clinical efficacy and feasibility need to be better disseminated across US primary care settings and its potential adopters. Future research is also needed to support the integration of HPV self-sampling within various delivery modalities (mail-based vs. clinic-based).

Contributions to the literature

- Human papillomavirus (HPV) self-sampling, an alternative cervical cancer screening strategy, has not been tested or implemented in the USA.
- This novel study captures considerations from US health care personnel on how to plan for the implementation of an HPV self-sampling practice.
- Several perceived multilevel barriers to HPV self-sampling were noted, including (1) at the institutional level, the need for additional clinic resources and education; (2) at the patient level, ensuring the validity of self-collected samples; and (3) at the test level, additional educational resources to facilitate the usability of test kits.
- While support for HPV self-sampling among health care personnel is growing, additional efforts are needed to disseminate the clinical efficacy and feasibility of this new screening tool in US primary care settings.

## Introduction

Persistent infections with high-risk human papillomavirus (HPV) types (e.g., 16, 18) are well-documented causes of cervical cancer [1]. In 2021, an estimated 14,480 new cases of cervical cancer will have occurred in the USA [2], resulting in health care costs associated with testing, treatment, and management of cervical malignancies [3]. As one of the most substantive medical expenditures for US women [4], wide-ranging public health efforts must be employed to address the burden of cervical cancer.

Since the implementation of cervical cancer screening (CCS) methods, such as cervical cytology (or “Pap test”), rates of cervical cancer have decreased [5–7]. However, cervical cancer continues to persist, and this burden is not distributed equally among all races and ethnicities. In 2020, Black/African-American and Hispanic/Latina women reported incidence rates of 8.4 cases per 100,000 women and 8.9 cases per 100,000 women, respectively [8]. Surveillance trends continue to point to these stark and ongoing cervical cancer disparities in minority groups. Most notably, Pap tests are still underutilized by many US minority women due to a lack of knowledge of cervical cancer and cervical cancer screening, psychosocial and cultural beliefs, pain associated with pelvic exams, and structural barriers to health care access [9–11].

Currently, US women aged 30–65 have three cervical cancer screening options based on clinic-based and clinician-obtained samples: (1) cervical cytology every 3 years, (2) a high-risk human papillomavirus (hrHPV) test every 5 years, or (3) a hrHPV testing in combination with cervical cytology (co-testing) every 5 years [12]. Notably, the US Preventive Services Task Force recommendation for hrHPV testing (primary HPV testing) was approved in 2018, with a recent similar guideline adoption by the American Cancer Society (ACS) in 2020 [13]. Primary HPV testing, which tests for the presence of an infection with a high-risk HPV type, is increasingly being incorporated into screening and follow-up guidelines for cervical cancer [14]. Nevertheless, health systems still experience slow adoption of primary HPV testing due to the recency of approved evidence-based guidelines [15].

HPV self-sampling, the process of collecting a vaginal sample by oneself for HPV testing, may help to mitigate some of the aforementioned barriers experienced by minority women and facilitate primary HPV testing within the USA [16]. Empirical studies examining the efficacy of HPV self-sampling have shown significant improvements in cervical cancer screening rates among women [17–20]. Some countries with organized cervical cancer screening programs (e.g., Great Britain) have already deployed HPV self-sampling as an adjunct strategy for increasing primary cervical cancer screening [20, 21]. HPV self-sampling has been shown to be effective in reaching women who otherwise delay or opt out of cervical cancer screening [17–21]. In studies examining acceptability and preference, many women report a high acceptability of HPV self-sampling, and in some cases, women indicated a higher preference for self-collected HPV tests than provider-collected tests due to their convenience and less invasiveness [22–25]. Multiple cost-effectiveness analyses have also found that HPV self-sampling had a lower lifetime cost and a higher quality-adjusted life expectancy than Pap test screening [26–28]. These findings suggest that HPV self-sampling is a potentially cost-saving and effective strategy to increase cervical cancer screening among women who may not readily undergo routine Pap tests. However, most research in this area has focused on mail-based self-sampling kits [17, 18], and an untapped opportunity exists to utilize clinic-based HPV self-sampling in health care systems [29]. Providing clinic-based HPV self-sampling may help resolve common issues related to the mailing of samples (e.g., missing samples) and patients’ questions about how to conduct the self-collection (e.g., visual tutorials provided by clinic staff).

To date, no planning or pre-implementation study has been conducted to assess if HPV self-sampling—whether as a mail-based or clinic-based approach—can be used to facilitate primary HPV testing in the USA. Furthermore, instituting a new practice or tool within health care settings requires a systems understanding of the contexts and actors involved in health care decisions and service provision [30]. Guided by the Consolidated Framework for Implementation Research (CFIR) [31], a multilevel framework, this qualitative study aimed to describe the perspectives of health systems leaders, primary care clinicians, and clinic staff on the possible implementation of an HPV self-sampling practice in primary care clinics.

## Methods

### Study population and procedures

Health care professionals across three personnel types—health systems leaders, providers, and clinic/lab staff—were recruited and interviewed for the study. Health systems leaders included individuals who were medical directors and operating officers embedded within a health system. Providers included primary care clinicians, general medical practitioners, obstetrics/gynecologists, nurse practitioners, and physician assistants employed in primary care settings. Clinic and/or lab staff included lab staff, nursing staff, and community health workers embedded within a primary care setting. Health care personnel were invited across various health care systems, including academic and community health centers, hospitals, and managed care organizations. A study invitation was emailed to the listserv of the health care professional networks across the Midwest, including urban and rural areas. The email invitation included information on the study, motivation of the research team, and compensation for completing the interview. A small number of participants (< 5) were also identified and recruited through snowball sampling. All participants were required to meet the following eligibility criteria: (1) read and write in English, (2) be 21 years or older, and (3) currently employed in a health care system or primary care setting. All interviews were video recorded along with field notes and conducted over Zoom for over 45–60 min with one or two female research team members (FH, RP, SX). Before each interview, verbal consent was obtained from all participating individuals. All participants were compensated with a mailed $50 gift card.

### Study measures and interview guide

All participants completed a short survey prior to their interviews. The survey, administered online through Qualtrics, assessed age, occupation, level of educational attainment, race/ethnicity, gender, and an awareness of HPV self-sampling (yes/no). The semi-structured interview guide was organized by three CFIR domains: intervention characteristics, inner setting, and process (Table 1). Questions around the intervention characteristics were intended to capture health care personnel’s perceptions of HPV self-sampling, including its advantages over Pap tests, its adaptability with clinic-based and/or home-based delivery approaches, and possible barriers to its implementation. If participants have not heard of HPV self-sampling, a description was provided to them at the time of the interview. Potential implementation considerations were also covered with questions in the latter two domains (inner setting, process). Before study recruitment, the interview guide was pilot tested with two primary care providers (not included in the study sample). All study protocols and materials were submitted for IRB approval and deemed exempt.

**Table 1:** Key informant interview guide and questions mapped onto CFIR domains

CFIR domain(s)	Core questions	Prompts
**Intervention characteristics—*evidence strength and quality; relative advantage***	1. What are your views on using self-collected HPV testing as a potential option for cervical cancer screening?	Would you like to have it as an option you could offer patients? Why or why not? Do you see any other particular advantages or disadvantages to HPV self-sampling?
**Intervention characteristics—*evidence strength and quality; relative advantage***	2. In your view, what do you think patients would think of self-collected HPV testing as an option for cervical cancer screening?	What about the patients who participate less?
**Intervention characteristics—*adaptability***	3. Do you think a mailed HPV self-sampling intervention would work within the context of your clinical setting? Why or why not?	What kinds of changes or alterations do you think will need to be made to mailed HPV self-sampling so that it will work effectively in your setting?
**Intervention characteristics—*adaptability***	4. HPV self-sampling could also be completed in the clinic during a clinic visit. Do you think an in-clinic HPV self-sampling intervention would work within the context of your clinical setting? Why or why not?	What kinds of changes do you think will need to be made to clinic-based HPV self-sampling so that it will work effectively in your setting?
**Inner setting—*HPV self-sampling awareness***	5. Have you heard about self-collected HPV testing as an option for cervical cancer screening?	
**Inner setting—*implementation climate*** **(subconstruct—compatibility)**	6. How well would a mail-based HPV self-sampling intervention fit with existing work processes and practices in your setting?	Can you describe how mail-based HPV self-sampling would best be integrated into current processes?
**Inner setting—*implementation climate*** **(subconstruct—compatibility)**	7. How well would a clinic-based HPV self-sampling intervention fit with existing work processes and practices in your setting?	Can you describe how clinic-based HPV self-sampling would best be integrated into current processes?
**Inner setting—*readiness for implementation*** **(subconstruct—access to knowledge and information)**	8. What kind of information do you think decision-makers would need if they were to consider implementing HPV self-sampling?	
**Process—*engaging*** **(subconstruct—opinion leaders)**	9. Who are the key decision makers that would influence if HPV self-sampling would be used in your clinical context?	Do you know if your health system has discussed adopting this new method for cervical cancer screening? Why or why not?

### Data analysis

Two researchers (NA, SX), both trained by a qualitative research expert (RP), independently coded a subset (10) of the interview transcripts. They double-coded the data to ensure consistency then met to discuss, review, and adjudicate any differences in coding. Coding differences were resolved by reviewing the data and developing a consensus on the central themes. A social constructivist grounded theory approach was used to identify meta-themes, themes, and subthemes in the data, allowing for the emergent findings to be loosely situated in the CFIR framework [32, 33]. Although the CFIR was used as a guiding framework, every attempt was made to retain the subjective meaning expressed by the respondents in the presentation of the study results. Ongoing discussions and consensus decision-making regarding the organizing codebook within the research team validated the rigor of the qualitative analysis. All interview data were organized, managed, and coded using the NVivo 12 software [34].

## Results

### Participants

Thirteen health care providers (*n* = 13, 43.3%), six clinic/lab staff (*n* = 6, 20.0%), and eleven health care leaders (*n* = 11, 36.7%) participated in the interviews (Table 2). The majority of participants were aged 40 and older (*n* = 19, 63.3%), non-Hispanic White (*n* = 25, 83.3%), female (*n* = 27, 90.0%), college-educated (*n* = 28, 93.3%), and had never heard of HPV self-sampling (*n* = 17, 56.6%). Most health care providers worked in an academic health center, whereas clinic/lab staff worked in community health centers, and health care leaders were predominantly from managed care organizations (e.g., payors, health plans).

**Table 2:** Study participant demographics (*N* = 30)

Sociodemographic variables	Health care providers (***N*** = 13), ***n*** (%)	Clinic/lab staff (***N*** = 6), ***n*** (%)	Health care leaders^**a**^ (***N*** = 11), ***n*** (%)	Total sample (***N*** = 30), ***n*** (%)
Age range				
18–29	–	1 (16.7)	**–**	1 (3.3)
30–39	3 (23.1)	3 (50.0)	1 (9.1)	7 (23.3)
40–49	7 (53.8)	1 (16.7)	6 (54.5)	14 (46.7)
50–59	2 (15.4)	–	1 (9.1)	3 (10.0)
60+	1 (7.7)	1 (16.7)	–	2 (6.7)
Race/ethnicity				
Non-Hispanic White	12 (92.3)	3 (50.0)	10 (90.9)	25 (83.3)
Black/African-American	1 (7.7)	1 (16.7)	–	2 (6.7)
Asian	–	2 (33.3)	–	2 (6.7)
Hispanic	–	–	1 (9.1)	1 (3.3)
Others	–	–	–	–
Gender				
Female	11 (84.6)	6 (100.0)	10 (90.9)	27 (90.0)
Health system type				
Academic health center	6 (46.1)	2 (33.3)	1 (9.1)	9 (30.0)
Community health center	4 (30.8)	4 (66.7)	3 (27.3)	11 (36.7)
Hospital-based system	3 (23.1)	–	1 (9.1)	4 (13.3)
Managed care organization	–	–	6 (54.5)	6 (20.0)
Highest level of education				
College Graduate	13 (100.0)	4 (66.7)	11 (100.0)	28 (93.3)
HPV self-sampling awareness				
No	3 (23.1%)	6 (100.0)	8 (72.7)	17 (56.7)

^a^Age data were not available for three health care leader respondents

### Intervention characteristics—relative advantages of HPV self-sampling

Compared to traditional cervical cancer screening (CCS) methods, participants reported many important potential advantages to offering HPV self-sampling within health care systems. At the institutional level, these benefits included increased reach and follow-up, especially among those who are traditionally underscreened due to personal barriers, such as limited English proficiency, low health literacy, and/or financial and structural barriers.

From a total population health management standpoint, it would have some advantages, especially when it’s targeted to groups with lower rates, in particular certain racial/ethnic minority groups or patients in specific demographics of gender minority, and patients who have the history of trauma who don’t feel comfortable with the [Pap test] procedure, but would feel comfortable with a tampon-like self-collection modality. In all likelihood, I think that it [HPV self-sampling] would be really high yield. (Leader)

Several respondents also perceived HPV self-sampling to be an important trauma-informed CCS tool, given the full control patients would be able to have over their own screening experiences.

Meanwhile, at the provider level, reducing stress and saving clinicians more time to conduct other clinical interactions were the most important advantages.

As the provider, I don’t like having to do some exams, if I don’t need to because it does take a lot of time to set up. It would be really nice to just be able to [have the patient] come in and do everything they needed to do. (Provider)

Respondents also reported perceived personal and procedural advantages to HPV self-sampling at the patient level. The most commonly cited personal advantages were ease of use, efficiency (i.e., not needing a provider to initiate the collection), comfort, privacy, and cost-saving (e.g., affordable test kits, fewer clinic visits). Some provider participants also perceived that HPV self-sampling could help to empower and cultivate diverse patients’ interest and proaction in their own health.

I think it would address a lot of concerns … for people of large sizes, different gender identities or racial and ethnic backgrounds. It would put the control over the testing literally in the hands of the patient. (Provider)

Some respondents, moreover, discussed significant procedural advantages to the self-sampling approach, such as its ability to mitigate invasiveness and pain and reduce time and burden for both patients and providers.

### Intervention characteristics—adaptability (e.g., advantages of mail-based vs. clinic-based approaches)

Specific advantages to the adaptability of HPV self-sampling—either within the context of a mail-based approach or a clinic-based approach—were also reported. The most important advantage to the mail-based approach was that it would be relatively easy to be integrated into existing workflows, but only if the implementing health care system has experience and success with past mailed campaigns and interventions.

We have in the past done some panel management where we’re going through and see where people are due for various things and then do a phone and mail outreach to try to get people to come in, etc. I could see that we could easily pull patients who are eligible or due for cervical cancer screening and mail them kits. (Leader)

Due to the recent COVID-19 pandemic, many health care systems had also pivoted to providing virtual care and telemedicine to their patients. Nearly all participants reported that their patient portals and electronic medical record (EMR) databases had been strengthened as a result. They noted that mailing HPV self-sampling kits would complement the telehealth services they were already offering to patients; the adoption of the mail-based approach, they perceived, would be ideal and feasible because it was leveraging an already extant infrastructure.

Clinic-based HPV self-sampling, on the other hand, was perceived by respondents to have an opportunistic advantage—that is, allowing patients to complete or take a self-sampling test kit while they are already at the clinic for other preventive care needs (such as sore throats or flu shots). Respondents working in health systems, where particular mailed campaigns have not been successful with certain patient populations (e.g., highly mobile), were most enthusiastic about this approach. Several provider participants shared that the clinic-based approach would ease some of their concerns about not having in-person/physical visits if the mail-based approach was instituted. They also noted that if any patient concerns around the self-collection arose at the clinic, they could address, advise, and troubleshoot those concerns in a timelier fashion than a mail-based approach.

It would be better if it’s done in the clinic where somebody can be there and support it, and answer any questions -- until women kind of get to the point where they have confidence and do it themselves. (Provider)

Additionally, the adoption of the clinic-based approach into existing workflows was perceived to be practical—as some clinicians stated, it would be similar to instituting lab orders (e.g., urine samples, blood draws) and could be completed within one clinic visit.

If you’re able to have lab staff do it, then the patient could come in anytime. And just like a urine sample. They could just go into the bathroom, do it, and then drop it off. (Lab staff)

### Inner setting—implementation climate

#### Awareness of HPV self-sampling

An overwhelming majority of respondents had not heard of HPV self-sampling. Once it was described, many of them perceived the tool favorably, particularly its potential to narrow racial and ethnic disparities in cervical cancer screening, and were excited to support the integration of it into their practices and health plans. Some providers also viewed HPV self-sampling as an alternative to traditional CCS tools; however, they did not perceive it as a primary screening tool.

I think an all-in approach is necessary to address some of these disparities and to have that [HPV self-sampling] as another tool, so that when somebody you know at the point of care, declines a Pap test, they can have another option. (Provider)

Despite the low level of awareness, a handful of providers had heard of HPV self-sampling and were already piloting the tool within their practices. Those who were piloting these services also reported positive support and satisfaction from many of their patients.

#### Barriers to HPV self-sampling

Most participants supported HPV self-sampling; however, many of them had reservations about the challenges to its implementation. These barriers were identified at the institutional, patient, and test levels. At the institutional level, the following challenges were noted: need for additional clinic resources (including the need to support follow-up care) and education of providers and patients, availability of test-kits, disruption to clinic workflows, interference with existing preventive care and routine practices, disjointed labs and clinics, lack of telehealth services, perceived lack of CCS rates and performances from partners and competitors, and negative past experiences with self-testing interventions.

At the patient level, the most commonly perceived barrier was the accuracy of self-collection. Several provider participants shared that patients’ perceptions and abilities to collect viable samples for HPV testing could be a potential hurdle to taking up HPV self-sampling effectively. They worried that (1) patients may believe that their self-collected samples will not be as sufficient or valid as a clinician-collected sample, preventing them from actually initiating the self-collection (perception) and (2) that patients may not be able to perform the self-collection accurately, resulting in samples that would not be conducive for analysis (ability).

The most important contributing factor to a patient’s ability to self-collect, as perceived by provider participants, was their self-efficacy—that is, having sufficient confidence, training/education, and comfort to collect a sample on their own even under constraints.

I think all women will have varying degrees of feeling comfortable with swabbing themselves. I could see people struggling with tampons, so I can see that being a challenge. I think just offering support and saying you don’t have to do this. This is just an option that we have now, and I can walk you through it, and you can try it out and I’m here to help you with it. I think that [support] could be really helpful, particularly the guidance of it. (Leader)

Most respondents believed that if patients were not trained to develop this individual capacity, they would likely not take up HPV self-sampling. Language access was also a reported barrier to establishing self-efficacy within patients. Provider participants shared that if multilingual educational resources around HPV self-sampling were not available, racial/ethnic groups could be disproportionately served. A few respondents suggested that having community health workers to assist and provide tutorials on the self-collection process can help mitigate this issue.

I think many of my BIPOC patients would be all for it but with the language barrier there’s just a need for a lot of continuing education. But once the idea is there, that you could just do HPV screening, it’ll be good. (Provider)

If someone did self-sampling a number of times and they felt comfortable doing it, you know, another thing we could even explore is a community health worker bringing it to somebody at their homes having them do it, and being kind of like on site and then taking it back, that might be another effective way of doing it as well. (Leader)

Several provider participants shared that older patients may be more resistant to HPV self-sampling—as it would require more training, counseling, and buy-in to get them on board with this tool. They believed that younger patients would be more likely to adopt this tool as they were perceived to be more mobile, having more time constraints, and exhibit lower learning curves. Some participants also mentioned that community support could be important to some racial and ethnic groups. For example, if some patients report positive attitudes and experiences with HPV self-sampling and share this information within their networks, other individuals within their communities may be more likely to initiate HPV self-sampling. Hence, a lack of community buy-in could be a potential barrier to widespread adoption.

There’s a lot of stigma for women who are getting/accessing health care services. Because of the different practices that happen in different communities, some women will rely on their peers, network to kind of inform her. (Leader)

Some participants also reported that HPV self-sampling may pose a challenge to patients with variable sexual anatomies, such as those with circumcisions and imperforate hymens. One provider respondent also shared that HPV self-sampling may not be appropriate for patients with physical disabilities, as the self-collection does require physical functioning to perform and collect vaginal samples.

I think about the mobility challenges potentially in someone who is postmenopausal with arthritis or other sorts of things that might make it more difficult for them to insert the swabbing. (Provider)

Some participants also perceived HPV self-sampling could create an additional patient burden. For example, if some patients were already experiencing challenges with coming into the clinic and navigating health care services, they would be less likely to initiate HPV self-sampling as it would require additional effort.

If a self-collection is going to not give us the cells and it has to be recollected then the patient is having to deal with that situation twice. So the disadvantage for that would be putting the patient in an even more uncomfortable position because now they’re having to deal with two swabs instead of one. (Lab staff)

Several respondents additionally shared that it could be challenging to motivate patients, who have a strong reliance and trusted relationship with their provider, to initiate HPV self-sampling—as these types of patients would much rather defer to traditional CCS methods that require clinician-collected samples.

At the test level, characteristics of HPV self-sampling test kits were also noted as potential barriers to implementation. Provider participants were most concerned with the level of evidence around the validity of self-collected samples. Many respondents cited that if self-collected samples were not as valid as clinician-collected samples, they would not support the use of the tool. Specifically, they had concerns about the possibility of self-collected samples creating false negatives and hesitancy around adopting HPV self-sampling if it had lower sensitivity than traditional CCS methods.

I wonder about the technicality of the test as far as its specificity and sensitivity. Is it as good when done within certain parameters you know? That’s one potential thing especially when it’s rolled out. (Provider)

Similarly, many participants had reservations about the potential for user errors. Respondents shared that if test kit instructions were too complicated, patients would be more likely to make missteps during the collection process. They suggested simplifying self-collection instructions into digestible formats (videos/illustrations) and instituting some feedback mechanisms (between patients and providers) that appropriately identified correctly collected samples.

It would be nice to have instructions in multiple languages, and maybe even the option of a video or a diagram. If there is like a contact person that could explain and just make sure that education is there, that would be helpful in supporting the patient. (Provider)

Other factors that could also contribute to user error included the types of self-collection instruments and their requirements for sample viability. The concerns raised by respondents about the collection instruments (e.g., brush) were based on their usability factors (i.e., perceived user-friendliness). Some participants believed that the lavage—the washing out of a body cavity—would be more difficult to use since it required several steps to collect and prepare a solution. Meanwhile, others thought that a brush could pose more challenges as it may feel more uncomfortable for some patients.

I think a big barrier could be the brush. If a patient can’t tolerate a bigger brush, she might not do it. Maybe if there is a guided path brush that is flat, that might be easier to do. (Provider)

Barriers to achieving sample viability were organized by the required composition of a viable sample and the procedural requirements to maintain a viable sample. Many respondents inquired about the amount and types of cells (cervix only, cervicovaginal, or others) required for a sufficient sample.

I do have some concerns with it. If it is a molecular test that doesn’t require a lot of cells. But does it require the patient to get the swab on the cervix? My concern then is if the patient will be able to get it up that far by themselves to get the right type and amount of cells? (Lab staff)

Regarding procedural requirements, several concerns were raised about the shelf life and ability of test kits to maintain sample viability during transport. Specific transport barriers included the ability to protect the integrity of samples in extreme temperatures, prevent contamination, and/or be stored for an extended period of time before testing.

Additional costs associated with the implementation of HPV self-sampling included HPV self-sampling test kits not being covered by National Breast and Cervical Cancer Early Detection Programs or health plans. Regulatory challenges were also noted. Provider participants, who were aware of HPV self-sampling, knew that the approach is not currently an approved standard of care; this regulation, they cited, remained the biggest barrier to adoption. Other test-level concerns included the lack of FDA-approved HPV self-sampling tests and the potential for overtesting or overscreening in patient populations.

#### Specific barriers to mail-based vs. clinic-based HPV self-sampling

Specific challenges were also noted about the clinic- and mail-adapted HPV self-sampling approaches. Two barriers were identified for the clinic-based approach: (1) if health systems contained a policy that prevented onsite self-collection (e.g., exam rooms and bathrooms are not deemed sanitary) and (2) if no infrastructure is available to support onsite collection (e.g., curtains in exam rooms). In contrast, three specific barriers to the mail-based approach were identified by participants. The concerns included costs, lack of privacy, and mailing logistics. Several respondents stated that if the mail-based approach is adopted, significant institutional funding would be required to coordinate mailing logistics and cover mailed kits (e.g., postage, envelopes). Another barrier to the mail-based approach is the lack of privacy within the homes of patients to perform the self-collection. Having no safe and privately available space for patients to conduct the self-collection may make it less likely that they complete the self-sampling. A few provider participants shared that the lack of resident/home privacy could also create a potential for patients to be stigmatized and shamed for sexual activity if people are able to identify that they were initiating HPV self-sampling (such as neighbors or parents opening the mailed test kits of patients).

Many people live in congregate or intergenerational households. Maybe with partners who may or may not always be supportive. So when the test gets sent out, is it confidential? Because even though it’s a screening tool, somebody might think it’s a pregnancy test or that there’s something wrong that you’ve got an infection, which could cause stigma. These are potential unintended side effects of mailing and how confidential they are, especially for people that don’t live by themselves or don’t want anyone to know what they are doing. (Provider)

Finally, many health care personnel discussed several challenges with the logistics of the mail-based approach (particularly within the context of past and unsuccessful mailed interventions). They shared that obtaining a consistent mailing address in highly mobile patient populations was often difficult. When test kits and clinic information were mailed to these patients, they had already moved, so the test kits were lost or returned to the clinic. In cases where mailed test kits did successfully reach the patients, they were often misplaced or lost by patients. Overwhelmingly, many respondents did not support the idea of blindly mailing out HPV self-sampling test kits to patients.

### Process—planning and engaging (e.g., decision-making)

Most respondents shared that their health systems operated on a hierarchical leadership model when it came to the process of approving and instituting a new EBP. Respondents shared that senior executive leaders—such as chief operating officers (COOs), chief medical officers (CMOs), chief health officers, or clinic/unit/department managers—were the most important decision-makers within their health systems. These leaders were primarily responsible for packaging and presenting all the information (evidence, cost-effectiveness, feasibility, implementation guide) to health system stakeholders about approving a new EBP. The curation of evidence for institutional approval often involved a collaborative effort between these leaders, their implementation staff, and payers. Implementation staff, identified as providers/clinicians, lab staff, and EMR personnel, were needed to provide insights and planning on the actual integration of the EBP. Meanwhile, health care personnel working within payor organizations, such as public health analysts and health plan/economic researchers, played an important role in building the business case (cost/benefits, setup of reimbursement rates) of the EBP. Once approval is secured, the key implementation staff will lead the scale out of the EBP. Several respondents shared that the duration of approval to integration can range from half a year to as long as 3 years.

### Readiness for implementation

Many participants shared that multiple sources of information and evidence were needed from senior health care leaders to package a convincing argument for the approval and adoption of HPV self-sampling. These included whether the EBP has been adopted by their local competitors and/or partners; been shown to be cost-effective; demonstrated clinical efficacy, feasibility, and effectiveness; been adopted and recommended as a screening strategy by national clinical guidelines (such as American College of Obstetricians and Gynecologists (ACOG), American Society for Colposcopy and Cervical Pathology (ASCCP), American Society of Clinical Oncology (ASCO), United States Preventive Services Task Force (USPSTF)) and local clinical guidelines; been approved by regulatory bodies (Clinical Laboratory Improvement Amendments (CLIA); US Food and Drug Administration (FDA)); and been instituted into payment plans (Centers for Medicare & Medicaid Services (CMS)) and clinic performance metrics (National Committee for Quality Assurance (NCQA), Healthcare Effectiveness Data and Information Set (HEDIS)). One surprising and novel criterion that some health systems are considering as part of their approval package was if the EBP had demonstrated efficacy in reducing racial/ethnic disparities in care.

## Discussion

This study provided an in-depth assessment of health care personnel’s perspectives on HPV self-sampling, using CFIR to guide the exploration of factors for its potential implementation within US primary care settings. These key informants identified numerous barriers and facilitators to the potential adoption of an HPV self-sampling practice. The barriers and facilitators related to a number of CFIR domains: intervention characteristics, inner setting, and process. Of most salience to the pre-planning context of HPV self-sampling, however, was the intervention characteristics; the other domains were less present across all interviews.

Barriers to traditional Pap test screening in minority women have been documented extensively in the literature. These include psychosocial issues such as embarrassment, pain, and discomfort associated with a pelvic exam and practical issues such as difficulty finding the time to have the test or an acceptable (e.g., gender-concordant) doctor [9, 10]. HPV self-sampling was perceived by health care personnel to be more advantageous than traditional Pap tests because of its ease of use, efficiency, comfortability, privacy, and cost- and time-saving benefits. Of most importance to health care personnel was the tool’s potential ability to increase cervical cancer screening uptake within underscreened populations (e.g., rural, individuals’ sexual traumas). Health care personnel who largely represented non-Hispanic Whites, in particular, shared strong support for HPV self-sampling as an alternative CCS tool to increase uptake within racial/ethnic groups. As demonstrated in many empirical studies, HPV self-sampling has been shown to effectively increase cervical cancer screening uptake in hard-to-reach and minority populations [35, 36].

Health care personnel, however, were less sure about implementing both clinic-based and home-based HPV self-sampling approaches and whether a combined strategy would be most effective. As shared by study participants, several disadvantages and advantages existed within both delivery contexts. Implementation barriers to the mail-based approach specifically included mailing and laboratory logistics, disruption to clinical workflows, additional follow-up and lack of linkage to health systems, and extra costs. Most of these implementation issues are not new and have been consistently reported in HPV self-sampling intervention studies [22, 36] and other mailed, self-service testing models (e.g., HIV self-testing, mailed fecal immunochemical tests) [37–40].

In contrast, fewer implementation barriers to the clinic-based approach were noted and related to whether health systems had policies and infrastructures to support onsite self-collections. The lack of perceived barriers reported for this approach may be a function of the limited research on clinic-based HPV self-sampling. To date, only one pilot study (i.e., ROSE 1.0) has implemented HPV self-sampling onsite in a primary care setting [29]. The preliminary results of this study found that engagement, completion of HPV self-sampling, and follow-up among women were high. The optimistic findings from this pilot study align with health care personnel’s perceived advantages of the clinic-based approach, such as its ability to institute higher compliance, lower user error rates, and quicker turnaround time.

Regardless of the delivery approach, convincing decision-makers and developing acceptable implementation strategies within a health care system remain key to advancing the adoption of HPV self-sampling. As demonstrated in prior studies, key decision-makers’ capacity to sell an intervention, provide support, spell out roles for key staff, and reflect on implementation progress are important for successful implementation [41, 42]. Information of most interest for key decision-makers, as perceived by health care personnel, included clinical efficacy (e.g., test characteristics of HPV self-sampling), feasibility, effectiveness, and adoption and recommendation of HPV self-sampling as a screening strategy by both national and local clinical guidelines. With the increased movement at the federal level to reduce disparities in care [43], some key decision-makers were also beginning to call for evidence of the intervention’s impact to reduce racial/ethnic disparities. Strategies that tailor to these leaders, therefore, should be included in any implementation plan to integrate HPV self-sampling within health care systems.

When implementing a complex and novel health care intervention like HPV self-sampling, it is important to understand and anticipate potential barriers and facilitators within the health care delivery system [30]. Conducting interviews with key actors in such systems prior to implementation and applying an implementation framework like the CFIR, can enable researchers and practitioners in health care systems to identify emergent issues and plan for such contingencies that may affect the successful uptake of HPV self-sampling. Overall, these findings have the potential to inform future cervical cancer screening priorities and practices, as well as add to the emerging body of evidence that uses CFIR to examine pre-implementation processes [44].

### Strengths and limitations

The strength of this study was the inclusion of various health care personnel to reveal several views. The use of the CFIR also helped to identify multilevel factors that could impede or facilitate the implementation success of an HPV self-sampling practice [31]. These results further contribute to the growing evidence and literature on CFIR applications within pre-implementation studies [44]. This study also carried with it the limitations of qualitative research, including limited generalizability due to the selectivity of the sample and the limited number of interviewees. To reduce participant burden within working health care professionals, not all CFIR domains were also examined (i.e., characteristics of individuals). The use of a semi-structured interview guide could have also introduced some investigator bias, which may have influenced the data collection, analyses, and interpretation. To minimize this issue, the research team did conduct some respondent validation (i.e., member checking) of the results with a subset of interviewees through oral presentations.

## Conclusion

Support for HPV self-sampling among US health care personnel is growing. However, the evidence of its clinical efficacy and feasibility needs to be more widely communicated and better disseminated to key decision-makers within US primary care systems to facilitate its adoption. Specific consideration around the intervention’s characteristics, such as its relative advantages over other traditional CCS methods, its clinical evidence and feasibility, and its adaptability are important components to include in this packaging of information to key decision-makers. Future research is also needed to examine the effectiveness of integrating HPV self-sampling within various delivery contexts (mail-based vs. clinic-based).

## Data Availability

The datasets used and/or analyzed during the current study are available from the corresponding author upon reasonable request.

## Abbreviations

ACOG: American College of Obstetricians and Gynecologists
ACIP: Advisory Committee on Immunization Practices
ACS: American Cancer Society
ASCCP: American Society for Colposcopy and Cervical Pathology
ASCO: American Society of Clinical Oncology
CCS: Cervical cancer screening
CDC: Centers for Disease Control and Prevention
CFIR: Consolidated Framework for Implementation Research
CLIA: Clinical Laboratory Improvement Amendments
CMO: Chief medical officer
CMS: Centers for Medicare & Medicaid Services
COO: Chief operating officers
COVID-19: Coronavirus disease 2019
EMR: Electronic medical record
EBP: Evidence-based practice/program/policy
FDA: US Food and Drug Administration
FQHC: Federally-Qualified Health Center
HEDIS: Healthcare Effectiveness Data and Information Set
hrHPV: High-risk human papillomavirus
HPV: Human papillomavirus
IRB: Institutional Review Board
NCQA: National Committee for Quality Assurance
USPSTF: United States Preventive Services Task Force
